# Nutritional Vulnerability: The Complexity of Preparing Older Veterans for Surgery

**DOI:** 10.1093/geroni/igaa057.2794

**Published:** 2020-12-16

**Authors:** Kathyrn Starr, Nancy Loyack, Shelley McDonald, Mitchell Heflin, Sandhya Lagoo-deenadayalan, Connie Bales

**Affiliations:** 1 Duke University School of Medicine, Durham, North Carolina, United States; 2 Durham VA Medical Center, Durham, North Carolina, United States; 3 Duke University, Durham, North Carolina, United States; 4 Duke University Medical Center, Durham, North Carolina, United States

## Abstract

Poor nutritional status leads to postoperative complications, infections, poor healing, and increased mortality, creating a high-risk situation for older adults undergoing surgery. Psychosocial and environmental factors, including socioeconomic disadvantage, disability, social isolation and depression, are known precipitators of nutritional risk. However, these potentially modifiable concerns are rarely taken into consideration preoperatively. In 736 older Veterans preparing for surgery, we found 42% reported it was hard/somewhat hard to pay for basic needs, 6% reported sometimes/often times not having enough to eat, 24% reported living along, 47% reported needing assistance with 1 or more IADLs, and 42% reported a history of depression. Findings from older, Veterans, illustrate the prevalence of psychosocial and environmental risk factors prior to surgery. Best practices for identifying these factors, the importance of an interprofessional team for intervening, and specific resources that can be utilized throughout the perioperative period to improve outcomes will be presented.

